# NOD2 (Nucleotide-Binding Oligomerization Domain-Containing Protein 2)-Mediated Modulation of the Immune Response Induced by BCG (Bacillus Calmette-Guérin) Bacilli

**DOI:** 10.3390/pathogens14070683

**Published:** 2025-07-11

**Authors:** Magdalena Jurczak, Joanna Kaczmarek, Magdalena Kowalewska-Pietrzak, Paulina Stelmach, Magdalena Druszczynska

**Affiliations:** 1 Department of Immunology and Infectious Biology, Institute of Microbiology, Biotechnology, and Immunology, Faculty of Biology and Environmental Protection, University of Lodz, Banacha 12/16, 90-237 Lodz, Poland; paulina.stelmach@edu.uni.lodz.pl (P.S.); magdalena.druszczynska@biol.uni.lodz.pl (M.D.); 2 The Bio-Med-Chem Doctoral School of the University of Lodz and Lodz Institutes of the Polish Academy of Sciences, 90-237 Lodz, Poland; 3 Regional Specialized Hospital of Tuberculosis, Lung Diseases and Rehabilitation in Lodz, Okolna 181, 91-520 Lodz, Poland; asiakaczmarek@op.pl (J.K.); magda.kp@interia.pl (M.K.-P.)

**Keywords:** Bacillus Calmette-Guérin, respiratory syncytial virus, severe acute respiratory syndrome coronavirus 2

## Abstract

The Bacillus Calmette-Guérin (BCG) vaccine confers broad, non-specific immunity that may bolster defenses against respiratory viruses. While NOD2 (nucleotide-binding oligomerization domain-containing protein 2)-driven pathways are central to innate immune responses, the contribution of surface receptor modulation on monocytes to shaping these responses remains underexplored. We analyzed whole-blood cultures from BCG-vaccinated Polish children, stratified by serostatus to SARS-CoV-2 and RSV, and stimulated for 48 h with live BCG, purified viral antigens, or both. RT-qPCR quantified mRNA levels of NOD2 and key cytokines (IL-1β, IL-2, IL-4, IL-6, IL-8, IL-10, TNF), while flow cytometry assessed CD14, HLA-DR, CD11b, and CD206 expression. Co-stimulation with BCG + RSV elicited the strongest transcriptional response, notably a 2–4-fold upregulation of NOD2, IL-1β, and IL-6 versus RSV alone. In SARS-CoV-2(+) donors, RSV alone induced higher NOD2 expression than BCG or BCG + RSV, while IL-2 peaked following BCG + SARS-CoV-2. Across conditions, NOD2 positively correlated with IL-4 and IL-6 but negatively correlated with IL-1β in SARS-CoV-2 cultures. Viral antigens increased CD14 and HLA-DR on monocytes, suggesting activation; CD206 rose only in dual-seropositive children. Our findings indicate that BCG stimulation affects pediatric antiviral immunity through NOD2-related cytokine production and induction of a CD14^+^HLA-DR^+^ phenotype, supporting its potential role in boosting innate defenses against respiratory pathogens.

## 1. Introduction

Bacille Calmette-Guérin (BCG) is a live attenuated vaccine containing *Mycobacterium bovis*, mainly used against tuberculosis, but also provides non-specific protection against various pathogens, including respiratory viruses [1]. This phenomenon is referred to as ‘trained immunity’, in which innate immune cells are reprogrammed, leading to an enhanced response to subsequent infection, mediated by various signaling pathways, including those involving nucleotide-binding oligomerization domain-containing protein 2 (NOD2) [2,3]. NOD2 recognizes muramyl dipeptide (MDP) of bacteria and ssRNA of viruses [4,5]. NOD2 can detect peptidoglycan fragments of mycobacteria, such as MDP, initiating an immune response, particularly in the context of infection and vaccination with BCG [6]. Importantly, the BCG vaccine has been shown to engage the NOD2 receptor, leading to the epigenetic reprogramming of monocytes, during which the histone modification of histone 3 at lysine 4 (H3K4me3) enhances the expression of pro- and anti-inflammatory cytokine genes [7]. In addition, metabolism is activated by the Akt/mTOR pathway, resulting in a change in metabolism to glycolysis [8]. This reprogramming enhances the production of cytokines such as tumor necrosis factor alpha (TNF-α) and interleukin-1 beta (IL-1β) during subsequent encounters with unrelated pathogens, which is a characteristic of trained immunity [1].

Studies suggest that BCG can enhance immune responses against influenza, human papillomavirus (HPV) and herpes simplex virus (HSV), respiratory syncytial virus (RSV), and severe acute respiratory syndrome coronavirus 2 (SARS-CoV-2) [9,10]. A recent study showed that mice previously vaccinated with BCG and then infected with the influenza virus and SARS-CoV-2 were better at eliminating viruses and showed less weight loss. This effect was linked to a two-step innate response and a strong type 1 helper T lymphocyte (Th1) response in the lungs. And the presence of CD4^+^ lymphocytes producing interferon-gamma (IFN-γ) was important for long-term antiviral immunity, demonstrating the synergy of innate and adaptive immunity in BCG-induced non-specific protection [9]. In addition, a recombinant BCG vaccine expressing the RSV nucleoprotein (rBCG-N-hRSV) has been developed and shows promising immunogenic and protective properties. This vaccine induces both cellular and humoral responses [10]. In animal models, the administration of rBCG-N-hRSV has been shown to prevent lung damage associated with RSV infection and reduce inflammation. The protective mechanism of the vaccine includes the early recruitment of CD4+ and CD8+ T lymphocytes to the lungs and increased secretion of the cytokines IFN-γ and IL-17, which play a key role in the control of infection [11].

Given the role of the NOD2 receptor in recognizing bacterial and viral components and orchestrating downstream immune responses, we hypothesize that whole-blood stimulation in BCG-vaccinated children leads to an enhanced immune response to viral antigens (RSV, SARS-CoV-2), driven by NOD2-dependent mechanisms. Specifically, we propose that BCG vaccination primes innate immune cells to mount a more robust and flexible antiviral response upon secondary exposure to viral antigens, potentially counteracting immune-evasion strategies employed by SARS-CoV-2 and RSV. This effect may be mediated through the upregulation of NOD2 expression and context-dependent modulation of inflammatory cytokine production, including IL-1β, IL-2, IL-4, and IL-6, ultimately supporting more effective viral clearance.

In our study, using an in vitro whole blood stimulation model, we analyze the immune responses in children vaccinated with BCG, taking into account their serological status against RSV and SARS-CoV-2. We further hypothesize that the modulation of monocyte surface markers such as CD14, HLA-DR, and CD206 contributes to this enhanced responsiveness. In addition, we are investigating whether previous exposure to viruses such as RSV or SARS-CoV-2 is associated with differential innate response characteristics in children after BCG vaccination. Elucidating these interactions may offer novel insights into innate immune reprogramming and support the development of BCG-based immunomodulatory strategies against respiratory viral infections in pediatric populations.

## 2. Materials and Methods

### 2.1. Study Population

The study included 48 healthy children between the ages of 6 and 12 who had received the *Mycobacterium bovis* BCG Moreau vaccine (Biomed, Lublin, Poland) on the first day of life, in accordance with the Polish national vaccination program. No deviations from the Polish National Immunization Program were noted, and no child had received additional non-mandatory or experimental vaccines beyond those included in the standard schedule. While it is recognized that some vaccines may exert non-specific immunomodulatory effects, the standardized nature of vaccination coverage across all participants in our study limits the likelihood that such differences could introduce meaningful bias. Since all children were comparably immunized according to national guidelines, any minor variation in timing or formulation (e.g., different vaccine brands) is unlikely to have had a systematic effect on the immunological markers that we assessed. The inclusion criteria were as follows: (I) age between 6 and 12 years at the time of enrolment, (II) documented receipt of BCG Moreau vaccine on the first day of life, in accordance with the Polish national immunization schedule, (III) general good health status, with no current or chronic illnesses, as confirmed by a pediatric infectious disease specialist and a provincial pediatric pulmonology consultant, and (IV) informed written consent obtained from legal guardians. The exclusion criteria were as follows: (I) history of chronic respiratory diseases, including asthma, cystic fibrosis, or a previous diagnosis of tuberculosis, (II) any form of immunodeficiency (primary or secondary), including children on immunosuppressive therapy, (III) acute infection or antibiotic therapy within 30 days prior to sample collection, (IV) previous hospitalization for severe RSV or SARS-CoV-2 infection, and (V) a lack of compliance with the Polish vaccination schedule or missing documentation of BCG immunization. Based on the serostatus against RSV and SARS-CoV-2, participants were classified into four groups: (1) RSV-seropositive (RSV(+)), (2) SARS-CoV-2-seropositive (SARS-CoV-2(+)), (3) RSV- and SARS-CoV-2-seropositive (RSV(+) SARS-CoV-2(+)), and (4) RSV- and SARS-CoV-2-seronegative (RSV(−) SARS-CoV-2(−)) (Table 1). The children’s health was assessed by infectious disease consultants, including the provincial representative for pediatric pulmonology employed at the Provincial Specialist Hospital for Tuberculosis, Lung Diseases and Rehabilitation in Lodz. The Research Ethics Committee of the Medical University of Lodz approved the study protocol (No RNN/122/22/KE). SARS-CoV-2 antibodies were assessed using the DiaSorin LIASON^®^ SARS-CoV-2 TrimericS IgG assay on the LIASON^®^ XL analyzer (DiaSorin, Stillwater, MN, USA), with a diagnostic cut-off of ≥33.8 BAU/mL for positivity. According to the manufacturer, this assay demonstrates 98.7% sensitivity (≥15 days post symptom onset) and 99.5% specificity, with no significant cross-reactivity reported for seasonal coronaviruses, influenza A/B, RSV, or adenovirus. RSV infection was confirmed using the Respiratory Syncytial Virus IgG ELISA kit (Serion-Diagnostics, Würzburg, Germany), calibrated to WHO standards. Results ≥16 U/mL were considered positive (8–16 U/mL equivocal, <8 U/mL negative). The test’s reported sensitivity and specificity are 96.2% and 98.4%, respectively, with <1% cross-reactivity with human metapneumovirus or parainfluenza viruses.

### 2.2. Whole-Blood Cultures

Whole blood (7 mL) was collected into heparinized tubes (Sarstedt, Germany) and maintained at room temperature. All samples were processed within 2 h of collection. Leukocyte viability was assessed using the trypan blue exclusion method, and only samples with ≥95% viable cells were included in the analysis. For stimulation, whole blood was plated directly into 24-well culture plates (Nunc, Denmark) at 1 mL per well without the prior separation of peripheral blood mononuclear cells. Cultures were incubated at 37 °C in a humidified atmosphere containing 5% CO_2_ for 48 h. The first involved the stimulation of blood cells with SARS-CoV-2 virus peptides (1 µg/well) (Peptivator, MiltenyiBiotec, Bergisch Gladbach, Germany), in combination with live *Mycobacterium bovis* BCG strain Moreau (Biomed, Lublin, Poland; 10^6^ cells per culture). These cultures were compared with cultures stimulated with SARS-CoV-2 peptides alone or with BCG mycobacteria alone. The second type of culture involved the stimulation of blood cells with RSV antigens (1 µg/well) (PepTivator RSV, MiltenyiBiotec, Germany), also in combination with live *M. bovis* BCG mycobacteria. Again, parallel control cultures were conducted in which cells were stimulated with RSV peptides alone or with mycobacteria BCG alone [12,13]. Control conditions included cultures stimulated with BCG alone, with viral peptides alone, or with phytohemagglutinin (PHA; 10 µg/well; Thermo Fisher Scientific, Waltham, MA, USA) and unstimulated cultures in RPMI-1640 medium (Sigma-Aldrich, Gillingham, UK). After 48 h, culture supernatants were collected, centrifuged (1000× *g* for 10 min), aliquoted, and stored at −80 °C until cytokine quantification. The cell cultures were also frozen at −80 °C and used for cytometric analysis at a later date. Samples with visible hemolysis or low viability (<90%) were excluded.

### 2.3. Isolation of DNA and Its Spectrophotometric Evaluation

DNA was isolated from peripheral blood collected from all volunteers using EDTA tubes and the BD Vacutainer^®^ blood collection system. Isolation was performed within no more than 2 h of sample collection using the commercial EXTRACTME DNA BLOOD KIT (Blirt, Gdansk, Poland). To 200 μL of blood, 200 μL of erythrocyte lysis buffer was added, and samples were centrifuged for 4 min at 8600× *g*. After centrifugation, the supernatant was carefully removed from the white cell pellet and the cells were washed with 375 μL of BL buffer. Then, 6 μL of Proteinase K was added, and samples were incubated at 55 °C for 10 min. After the incubation was completed, 400 μL of buffer BB was added and the entire lysate was transferred to a DNA purification column. The samples were centrifuged for 60 s at 11,000× *g*. The column was then transferred to a new collection tube and washed twice: first with 600 μL of buffer BW1 and then with 400 μL of buffer BW2, each time centrifuging for 30 s at 11,000× *g*. In order to completely remove the alcohol from the column before elution, the samples were subjected to an additional centrifugation for 2 min at 21,000× *g*. Finally, DNA was eluted by adding 50 μL of elution buffer directly to the membrane of the purification column, incubating for 2 min and centrifuging for 60 s at 11,000× *g*. BioDrop µLITE (Holliston, MA, USA) was used to assess the quality and quantity of isolated DNA. The extracted DNA was stored at −80 °C until analyzed.

### 2.4. Assessment of the NOD2 Gene Polymorphism

To determine the genotypes, the isolated DNA was analyzed via PCR-RFLP. For the determination of R702W (rs2066844) variants, the following primer pairs were used: 5′-AGATCACAGCAGCCTTCCTG-3′, 5′-CACGCTCTCTTGGCCTCACC-3′. The 185 bp PCR product was digested at 37 °C for 1 h with 2 U MspI (EURx). The following fragments could be obtained by obtaining the following fragments: 20, 35, 54, and 76 bp in R702R homozygotes; 20, 35, 54, 76, and 130 bp in R702W heterozygotes; and 20, 35, and 130 bp in W702W homozygotes [14]. For the G908R (rs2066845) mutation assay, the primers 5′-CTCTTGGCCTTTCAGATTCTG-3′ and 5′-CAGCTCCTCCCTCTTCACCT-3′ were used. The 163 bp PCR product was digested at 37 °C for 1 h with 2 U HhaI (EURx). The following fragments could be obtained by obtaining the following fragments: 163 bp in G908G homozygotes; 27, 136, and 163 in G908R heterozygotes; and 27 and 136 bp in R908R homozygotes [14]. For the 1007fs (rs5743293) mutation assay, the primer pairs 5′-GGCAGAAGCCCTCCTGCAGGCC-3′ and 5′-CCTCAAAATTCTGCCATTCC-3′ were used. The 151 bp PCR product was digested for 1 h at 24 °C with 2 U ApaI (EURx). The following fragments could be obtained by obtaining the following fragments: 151 bp for Leu1007Leu homozygotes; 20, 131, and 151 bp in Leu1007Pro heterozygotes; and 20 and 131 bp in Pro1007Pro homozygotes [14].

A gradient thermocycler (BioRad, Hercules, CA, USA) was used to determine appropriate reaction temperatures and primer concentrations. The PCR reaction conditions are shown in Table 1. To visualize the digestion products (R702W, G908R), electrophoresis was run in a 12% polyacrylamide gel, and, for the 1007fs mutation, electrophoresis was run in a 2% agarose gel in TBE buffer at 70 V for 90 min, and the image was then documented on a ProXima 2750 system (Isogen Life Science, Utrecht, The Netherlands).

### 2.5. Isolation of RNA and Its Spectrophotometric Evaluation

The QIAamp RNA Blood Mini Kit (Qiagen, Hilden, Germany) was used to isolate RNA from culture sediments. For erythrocyte lysis, 1 mL of the cultured pellet was mixed with 5 mL of EL buffer and incubated at 4 °C for 15 min. After incubation, the cells were centrifuged for 10 min at 400× *g* at 4 °C. The pellet was then resuspended in 2 mL of EL buffer and centrifuged again for 10 min at 400× *g* at 4 °C. The resulting leukocyte pellet was resuspended in 350 μL of RTL buffer, which disrupts the cells. The entire lysate was transferred onto a QIAshredder column and centrifuged at maximum speed for 2 min to ensure homogenization. Subsequently, 350 μL of 70% ethanol was added to the homogenized lysate and carefully transferred onto a QIAamp column, followed by centrifugation for 15 s at 8000× *g*. The column was washed twice: first with 700 μL of RW1 buffer, then with 500 μL of RPE buffer, each time centrifuged for 15 s at 8000× *g*. An additional 500 μL of RPE buffer was added, and the sample was centrifuged for 3 min at 20,000× *g*. To completely remove any remaining RPE buffer, the column was subjected to an additional centrifugation step for 1 min at 20,000× *g*. Finally, RNA was extracted by adding 30 μL of RNase-free water directly onto the column and centrifuging for 1 min at 8000× *g*. To assess the quality and concentration of the extracted nucleic acids, RNA levels were measured using a BioDrop µLITE spectrophotometer (Holliston, MA, USA). A water sample free of the analyzed substances was used for calibration. Additionally, RNA integrity was assessed via electrophoresis on a 1.2% agarose gel in TAE buffer, run at 90 V for 60 min. The Gel-Doc 2000 system (Bio-Rad, Hercules, CA, USA) and Quantity One software (Version 4.6.8) were used to document gel images. The extracted DNA was stored at −80 °C until analyzed.

### 2.6. Reverse Transcription

A commercial iScript™ cDNA Synthesis Kit (Bio-Rad, Hercules, CA, USA) was used for cDNA synthesis. The synthesis of cDNA was performed according to the manufacturer’s instructions, using 1 μg of previously assessed RNA for integrity and quality as a template. Reverse transcription was performed using a Biometra UNO II thermocycler (Analytik, Jena, Germany), using parameters according to the kit manufacturer’s instructions. The resulting cDNA was stored at −20 °C until analysis.

### 2.7. qPCR Reaction

The reaction mixture for the IL-1B, IL-8, TNF, and IL-10 primers (BioRad, Hercules, CA, USA) contained 5 μL of iTaq universal SYBR Green Supermix, 0.5 μL of primer (20 μM), 3 μL of nuclease-free water, and 1 μL of cDNA, while for IL-2, IL-4, and IL-6 primers, it contained 5 μL of iTaq universal SYBR Green Supermix, 0.5 μL of each primer (25 μM), 3 μL of nuclease-free water, and 1 μL of cDNA (Table 2). Amplification was performed using the CFX96 Real-Time PCR Detection System (Bio-Rad, Hercules, CA, USA). The protocol included a pre-activation step at 95 °C for 2 min, followed by 40 cycles comprising denaturation (95 °C for 5 s) and annealing and elongation (60 °C for 30 s). An additional dissociation step was used to analyze the melting curve: initially, 65 °C for 5 s, followed by a temperature increase of 0.5 °C every 5 s until 95 °C was reached. GAPDH and HPRT1 were selected as reference genes due to their lowest M values, which indicate high expression stability. This makes them reliable internal controls, enabling precise normalization of the results [15]. The qRT-PCR reactions were performed in three technical replicates. The ΔΔCt method was used to analyze gene expression levels, enabling the assessment of relative amounts of selected mRNA transcripts. This method involves comparing the expression of the target gene to that of a reference gene, using cycle threshold (Ct) values obtained from the qPCR reaction. In the first step, Ct values were determined for both target and reference genes in the tested samples, followed by the calculation of differences between these values (ΔCt).

### 2.8. Peripheral Blood Leukocyte (PBL) Harvest and Flow Cytometry

After 48 h of whole blood culture, culture supernatants were removed and EDTA in PBS (2 mM) was added to each well to pellet adherent cells. The cells were then incubated with 5 times the volume of RBCL lysis buffer (A&A biotechnology, Gdańsk, Poland) on ice for 15 min. After erythrocyte lysis, PBLs were harvested and resuspended in fetal bovine serum with 10% dimethylsulfoxide and frozen in liquid nitrogen. For cytometric analysis, cells were thawed in a water bath at 37 °C, and then, warm RPMI 1640/10%FBS/EDTA (2 mM) was added to wash out DMSO and detach adherent cells. PBLs were washed in warm RPMI 1640/10%FBS and incubated for 1 h for cell regeneration. After incubation, cells were harvested and resuspended in HBSS/1%BSA and stained for 30 min at 4 °C with the following antibodies: CD14-FITC; HLA-DR-Bv605; CD-11b-APC; CD206-PE (all from BD Biosciences, Franklin Lakes, NJ, USA). The measurement was performed using a FAC Symphony™ A1 (BD Biosciences, Franklin Lakes, NJ, USA). Compensation was performed using BD™ CompBead Plus (BD Biosciences, Franklin Lakes, NJ, USA) compensation particles stained separately with each antibody conjugate.

### 2.9. Statistical Analysis

The correlation between mRNA expression levels and NOD2 protein concentrations was assessed using Spearman’s nonparametric rank correlation test. Results with a *p*-value less than 0.05 were considered statistically significant. Due to multiple comparisons, the Benjamini-Hochberg procedure was used to control the false discovery rate (FDR). The correction was performed globally, i.e., collectively for all tests performed as part of the cytokine analysis, which allowed for consistent control of the risk of false positive results across the entire set of analyses. Results with an adjusted *p*-value < 0.05 were considered statistically significant. A post-hoc power analysis was performed using GPower 3.1 [17]. A one-way ANOVA was used to assess statistical power across the four independent groups: RSV(+) (n = 14), SARS-CoV-2(+) (n = 17), RSV(+)SARS-CoV-2(+) (n = 11), and RSV(−)/SARS-CoV-2(−) (n = 6), with a total sample size of N = 48. Assuming a medium effect size (f = 0.25) and a significance level of α = 0.05, the achieved statistical power was 0.69. These results indicate limited power to detect moderate between-group differences and a potential risk of type II errors, especially in comparisons involving the smallest group. All statistical analyses were performed using GraphPad Prism version 8 (GraphPad Software, La Jolla, CA, USA). FlowJo™ 10.2 software (FlowJo LLC, Beaverton, OR, USA) was used to analyze the flow cytometry data after prior generation of the compensation matrix. Monocytes were selected based on the expression of CD14 markers and their characteristics in terms of size and granularity.

## 3. Results

### 3.1. Assessment of the NOD2 Gene Polymorphism

The distribution of the G908R, R702W, and 1007fs (Figures S1–S3) genotypes in the study group is presented in Table 3. The R702R homozygous variant was observed in 100% of the children studied, as was the case for G908G and Leu1007Leu, suggesting that these variants may be predominant in the Polish pediatric population. The high frequency of homozygosity for R702R, G908G, and Leu1007Leu suggests the potential genetic stability of these variants within the Polish pediatric population.

### 3.2. mRNA Expression of NOD2

**Figure 1:**
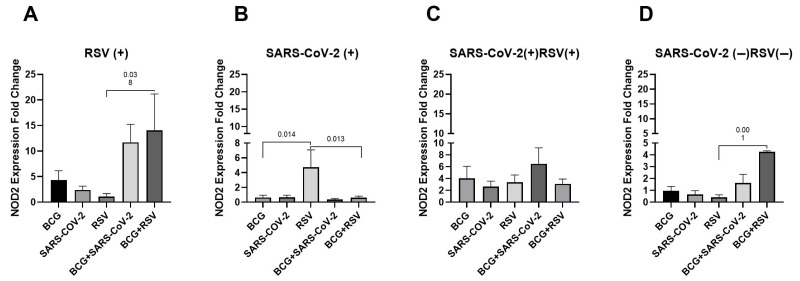
Changes in NOD2 gene mRNA expression in different groups of subjects who were simultaneously stimulated with BCG bacilli and viral antigens. The data presented in the graphs reflect differences in cytokine levels between stimulated and unstimulated cultures. The *Y*-axis shows the relative change in expression compared to the control, expressed as a multiple relative to the unstimulated sample; NOD2, nucleotide-binding oligomerization domain containing 2; (**A**). RSV(+), group seropositive for RSV infection; (**B**). SARS-CoV-2(+), group seropositive for SARS-CoV-2; (**C**). RSV(+)SARS-CoV-2(+), group seropositive for RSV and SARS-CoV-2; (**D**). RSV(−)SARS-CoV-2(−), group seronegative for RSV and SARS-CoV-2; BCG, bacillus Calmette-Guérin; SARS-CoV-2, severe acute respiratory syndrome coronavirus 2; RSV, respiratory syncytial virus. To compare NOD2 concentrations between individual study groups, a two-factor nonparametric Kruskal-Wallis analysis of variance was used, supplemented by Dunn’s post hoc test. Results with a *p*-value lower than 0.05 were considered statistically significant.

Figure 1 demonstrates a comparison of NOD2 gene mRNA expression in cultured whole blood. The highest NOD2 mRNA expression was observed in the RSV(+) and RSV(−)/SARS-CoV-2(−) groups co-stimulated with *Mycobacterium bovis* BCG and RSV antigens (14.02 ± 17.83 and 4.27 ± 0.09, respectively). In RSV-seropositive and RSV-seronegative groups, as well as in SARS-CoV-2 groups, a statistically significant increase in NOD2 gene expression was observed in cells co-stimulated with BCG and RSV antigens, compared to cells stimulated with RSV antigens alone. Furthermore, in the SARS-CoV-2-seropositive group, *NOD2* expression was significantly higher in cultures stimulated with RSV antigens alone compared to those stimulated with BCG alone or with the combination of BCG and RSV antigens alone.

### 3.3. mRNA Expression of IL-1β

**Figure 2:**
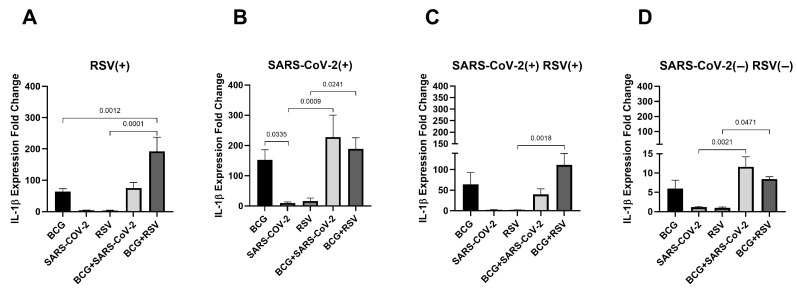
Changes in IL-1β gene mRNA expression in different groups of subjects who were simultaneously stimulated with BCG bacilli and viral antigens. The data presented in the graphs reflect differences in cytokine levels between stimulated and unstimulated cultures. The Y-axis shows the relative change in expression compared to the control, expressed as a multiple relative to the unstimulated sample; (**A**). RSV(+), group seropositive for RSV infection; (**B**). SARS-CoV-2(+), group seropositive for SARS-CoV-2; (**C**). RSV(+)SARS-CoV-2(+), group seropositive for RSV and SARS-CoV-2; (**D**). RSV(−)SARS-CoV-2(−), group seronegative for RSV and SARS-CoV-2, BCG, bacillus Calmette-Guérin, SARS-CoV-2, severe acute respiratory syndrome coronavirus 2; RSV, respiratory syncytial virus. To compare IL-1β concentrations between individual study groups, a two-factor nonparametric Kruskal-Wallis analysis of variance was used, supplemented by Dunn’s post hoc test. Results with a *p*-value lower than 0.05 were considered statistically significant.

Figure 2 illustrates the mRNA expression levels of IL-1β in cultured cell sediments. In the RSV(+) and SARS-CoV-2(+) RSV(+) groups, the highest IL-1β mRNA expression levels were observed in cultures stimulated with both *Mycobacterium bovis* BCG and RSV antigens (156.71 ± 149.06; 111.29 ± 78.81, respectively). In contrast, in the SARS-CoV-2(+) and SARS-CoV-2(−)/RSV(−) groups, the highest expression levels were detected in those co-stimulated with BCG and SARS-CoV-2 antigens (227.69 ± 204.67; 11.6 ± 4.44, respectively). A significant increase in IL-1β expression was observed in all study groups after co-stimulation with BCG and RSV antigens, compared to groups stimulated with RSV antigen alone. Conversely, in the SARS-CoV-2(+) and SARS-CoV-2(−)/RSV(−) groups, IL-1β mRNA expression was significantly higher in those co-stimulated with BCG and SARS-CoV-2 antigens compared to those stimulated with SARS-CoV-2 antigens alone. Additionally, a significant difference in IL-1β mRNA levels was observed in the RSV(+) group between cultures stimulated with BCG alone versus those co-stimulated with BCG and RSV antigens. Furthermore, a significant difference in IL-1β expression was noted in the SARS-CoV-2-seropositive group between cultures stimulated with BCG alone and those stimulated with BCG and SARS-CoV-2 antigens.

### 3.4. mRNA Expression of IL-8

**Figure 3:**
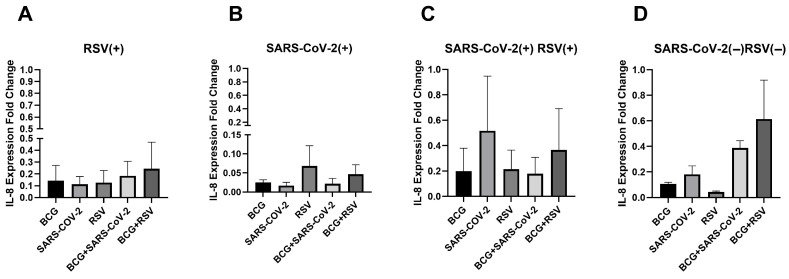
Changes in IL-8 gene mRNA expression in different groups of subjects who were simultaneously stimulated with BCG bacilli and viral antigens. The data presented in the graphs reflect differences in cytokine levels between stimulated and unstimulated cultures. The Y-axis shows the relative change in expression compared to the control, expressed as a multiple relative to the unstimulated sample; (**A**). RSV(+), group seropositive for RSV infection; (**B**). SARS-CoV-2(+), group seropositive for SARS-CoV-2; (**C**). RSV(+)SARS-CoV-2(+), group seropositive for RSV and SARS-CoV-2; (**D**). RSV(−)SARS-CoV-2(−), group seronegative for RSV and SARS-CoV-2, BCG, bacillus Calmette-Guérin, SARS-CoV-2, severe acute respiratory syndrome coronavirus 2; RSV, respiratory syncytial virus. To compare IL-8 concentrations between individual study groups, a two-factor nonparametric Kruskal-Wallis analysis of variance was used, supplemented by Dunn’s post hoc test. Results with a *p*-value lower than 0.05 were considered statistically significant.

Figure 3 depicts the mRNA expression levels of IL-8 in cultured cell sediments across all study groups. The highest IL-8 mRNA expression was observed in the SARS-CoV-2(−)/RSV(−) group in co-stimulated with both *Mycobacterium bovis* BCG and RSV antigens. However, no significant differences in IL-8 mRNA expression were detected between culture conditions in any of the study groups. 

### 3.5. mRNA Expression of IL-4

**Figure 4:**
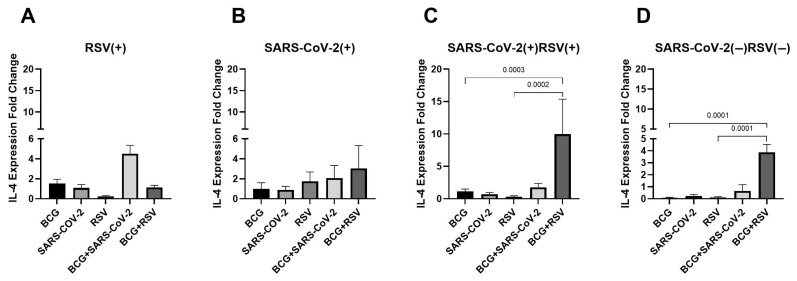
Changes in IL-4 gene mRNA expression in different groups of subjects who were simultaneously stimulated with BCG bacilli and viral antigens. The data presented in the graphs reflect differences in cytokine levels between stimulated and unstimulated cultures. The Y-axis shows the relative change in expression compared to the control, expressed as a multiple relative to the unstimulated sample; (**A**). RSV(+), group seropositive for RSV infection; (**B**). SARS-CoV-2(+), group seropositive for SARS-CoV-2; (**C**). RSV(+)SARS-CoV-2(+), group seropositive for RSV and SARS-CoV-2; (**D**). RSV(−)SARS-CoV-2(−), group seronegative for RSV and SARS-CoV-2, BCG, bacillus Calmette-Guérin, SARS-CoV-2, severe acute respiratory syndrome coronavirus 2; RSV, respiratory syncytial virus. To compare IL-4 concentrations between individual study groups, a two-factor nonparametric Kruskal-Wallis analysis of variance was used, supplemented by Dunn’s post hoc test. Results with a *p*-value lower than 0.05 were considered statistically significant.

As shown in Figure 4, IL-4 mRNA expression levels in cultured cell sediments were analyzed across all study groups. The highest expression levels were observed in those co-stimulated with both *Mycobacterium bovis* BCG and RSV antigens, in both seropositive and seronegative groups for RSV and SARS-CoV-2 (9.96 ± 9.33; 3.88 ± 0.66, respectively). These levels were significantly higher compared to cultures stimulated with RSV antigens alone or BCG alone. In contrast, no significant differences in IL-4 expression levels were detected between the RSV(+) and SARS-CoV-2(+) groups.

### 3.6. mRNA Expression of TNF

**Figure 5:**
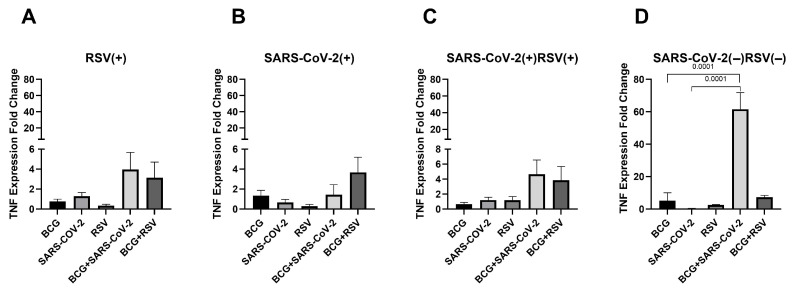
Changes in TNF gene mRNA expression in different groups of subjects who were simultaneously stimulated with BCG bacilli and viral antigens. The data presented in the graphs reflect differences in cytokine levels between stimulated and unstimulated cultures. The Y-axis shows the relative change in expression compared to the control, expressed as a multiple relative to the unstimulated sample; (**A**). RSV(+), group seropositive for RSV infection; (**B**). SARS-CoV-2(+), group seropositive for SARS-CoV-2; (**C**). RSV(+)SARS-CoV-2(+), group seropositive for RSV and SARS-CoV-2; (**D**). RSV(−)SARS-CoV-2(−), group seronegative for RSV and SARS-CoV-2, BCG, bacillus Calmette-Guérin, SARS-CoV-2, severe acute respiratory syndrome coronavirus 2; RSV, respiratory syncytial virus. To compare TNF concentrations between individual study groups, a two-factor nonparametric Kruskal-Wallis analysis of variance was used, supplemented by Dunn’s post hoc test. Results with a *p*-value lower than 0.05 were considered statistically significant.

Figure 5 illustrates the changes in TNF mRNA expression levels in cultured cell sediments. In the group seronegative for SARS-CoV-2 and RSV, the highest TNF mRNA expression levels were observed in cultures stimulated with both *Mycobacterium bovis* BCG and SARS-CoV-2 antigens (61.53 ± 10.31). Only in this group were statistically significant differences in TNF mRNA expression observed between cultures stimulated simultaneously with BCG and SARS-CoV-2 and those exposed to one of these antigens. No such differences were observed in other groups.

### 3.7. mRNA Expression of IL-10

**Figure 6:**
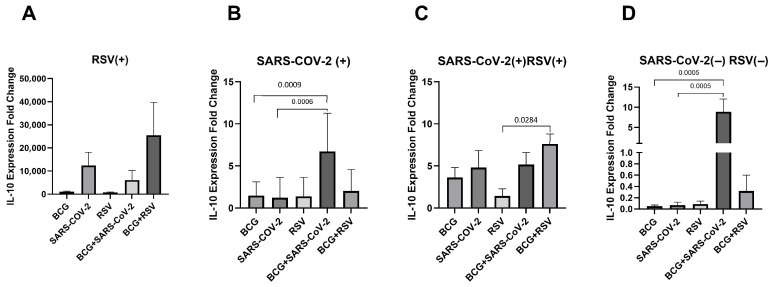
Changes in IL-10 gene mRNA expression in different groups of subjects who were simultaneously stimulated with BCG bacilli and viral antigens. The data presented in the graphs reflect differences in cytokine levels between stimulated and unstimulated cultures. The Y-axis shows the relative change in expression compared to the control, expressed as a multiple relative to the unstimulated sample; (**A**). RSV(+), group seropositive for RSV infection; (**B**). SARS-CoV-2(+), group seropositive for SARS-CoV-2; (**C**). RSV(+)SARS-CoV-2(+), group seropositive for RSV and SARS-CoV-2; (**D**). RSV(−)SARS-CoV-2(−), group seronegative for RSV and SARS-CoV-2, BCG, bacillus Calmette-Guérin, SARS-CoV-2, severe acute respiratory syndrome coronavirus 2; RSV, respiratory syncytial virus. To compare IL-10 concentrations between individual study groups, a two-factor nonparametric Kruskal-Wallis analysis of variance was used, supplemented by Dunn’s post hoc test. Results with a *p*-value lower than 0.05 were considered statistically significant.

Figure 6 presents the mRNA expression levels of IL-10 in cultured cell sediments. In the SARS-CoV-2(+) and SARS-CoV-2(−)/RSV(−) groups, the highest IL-10 mRNA expression was observed in cells co-stimulated with both *Mycobacterium bovis* BCG and SARS-CoV-2 antigens (6.27 ± 4.53; 8.86 ± 4.49, respectively). In the case of the other research groups, the highest level of expression occurred after the simultaneous use of BCG and RSV antigens (7.81 ± 3.48). A significant increase in IL-10 expression was observed in those co-stimulated with both BCG and SARS-CoV-2 antigens compared to those stimulated with either SARS-CoV-2 or BCG alone in the SARS-CoV-2(+) and SARS-CoV-2(−)/RSV(−) groups. Significantly higher IL-10 expression was detected in the co-stimulated condition (BCG + RSV) than with the RSV-only stimulation, specifically in the group seropositive for both viruses.

### 3.8. mRNA Expression of IL-6

**Figure 7:**
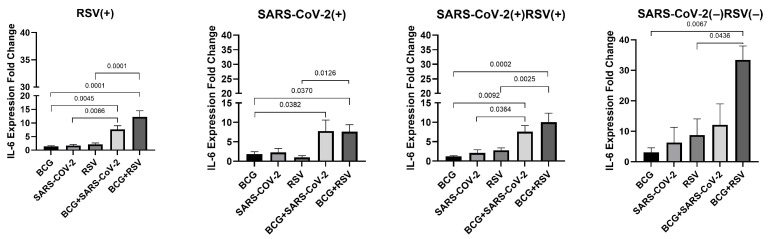
Changes in IL-6 gene mRNA expression in different groups of subjects who were simultaneously stimulated with BCG bacilli and viral antigens. The data presented in the graphs reflect differences in cytokine levels between stimulated and unstimulated cultures. The Y-axis shows the relative change in expression compared to the control, expressed as a multiple relative to the unstimulated sample; RSV(+), group seropositive for RSV infection; SARS-CoV-2(+), group seropositive for SARS-CoV-2; RSV(+)SARS-CoV-2(+), group seropositive for RSV and SARS-CoV-2; RSV(−)SARS-CoV-2(−), group seronegative for RSV and SARS-CoV-2, BCG, bacillus Calmette-Guérin, SARS-CoV-2, severe acute respiratory syndrome coronavirus 2; RSV, respiratory syncytial virus. To compare IL-6 concentrations between individual study groups, a two-factor nonparametric Kruskal-Wallis analysis of variance was used, supplemented by Dunn’s post hoc test. Results with a *p*-value lower than 0.05 were considered statistically significant.

Figure 7 illustrates the changes in IL-6 mRNA expression across all study groups. In the RSV(+), SARS-CoV-2(+)/RSV(+), and SARS-CoV-2(−)/RSV(−) groups, the highest IL-6 expression levels were observed in cultures stimulated with both *Mycobacterium bovis* BCG and RSV antigens (12.24 ± 7.99; 10.00 ± 7.33; 33.46 ± 6.41, respectively). In all study groups, IL-6 mRNA expression was significantly higher in BCG+RSV co-stimulated groups compared to cultures stimulated with either RSV antigens or BCG alone. Furthermore, in the RSV(+) and SARS-CoV-2(+)/RSV(+) groups, a significant increase in IL-6 mRNA expression was observed in BCG+SARS-CoV-2 co-stimulated groups compared to those stimulated with either SARS-CoV-2 antigens or BCG alone.

### 3.9. mRNA Expression of IL-2

**Figure 8:**
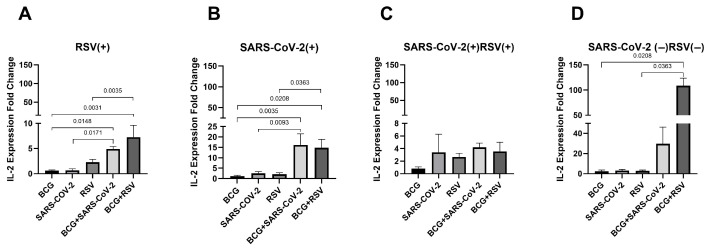
Changes in IL-2 gene mRNA expression in different groups of subjects who were simultaneously stimulated with BCG bacilli and viral antigens. The data presented in the graphs reflect differences in cytokine levels between stimulated and unstimulated cultures. The Y-axis shows the relative change in expression compared to the control, expressed as a multiple relative to the unstimulated sample; (**A**). RSV(+), group seropositive for RSV infection; (**B**). SARS-CoV-2(+), group seropositive for SARS-CoV-2; (**C**). RSV(+)SARS-CoV-2(+), group seropositive for RSV and SARS-CoV-2; (**D**). RSV(−)SARS-CoV-2(−), group seronegative for RSV and SARS-CoV-2, BCG, bacillus Calmette-Guérin, SARS-CoV-2, severe acute respiratory syndrome coronavirus 2; RSV, respiratory syncytial virus. To compare IL-2 concentrations between individual study groups, a two-factor nonparametric Kruskal-Wallis analysis of variance was used, supplemented by Dunn’s post hoc test. Results with a *p*-value lower than 0.05 were considered statistically significant.

Figure 8 illustrates the changes in IL-2 mRNA expression levels across all study groups. The highest expression levels were observed in the RSV(+) and SARS-CoV-2(−)/RSV(−) groups in cultures stimulated with both *Mycobacterium bovis* BCG and RSV antigens (7.27 ± 7.09; 108.63 ± 26.28, respectively), while in the SARS-CoV-2-seropositive group, the highest expression was detected in BCG+SARS-CoV-2 co-stimulated groups (16.15 ± 17.74). In the RSV(+), SARS-CoV-2(+), and SARS-CoV-2(−)/RSV(−) groups, IL-2 mRNA expression was significantly higher in BCG+RSV co-stimulated groups compared to those stimulated with either BCG or RSV antigens alone. Additionally, in the RSV(+) and SARS-CoV-2(+) groups, IL-2 mRNA expression was significantly elevated in BCG+SARS-CoV-2 co-stimulated groups compared to cultures stimulated with either BCG alone or SARS-CoV-2 antigens alone.

### 3.10. Relationship Between NOD2 and Cytokine Expression in Whole Blood Cultures Stimulated with Mycobacterium bovis BCG and Viral Antigens

**Figure 9:**
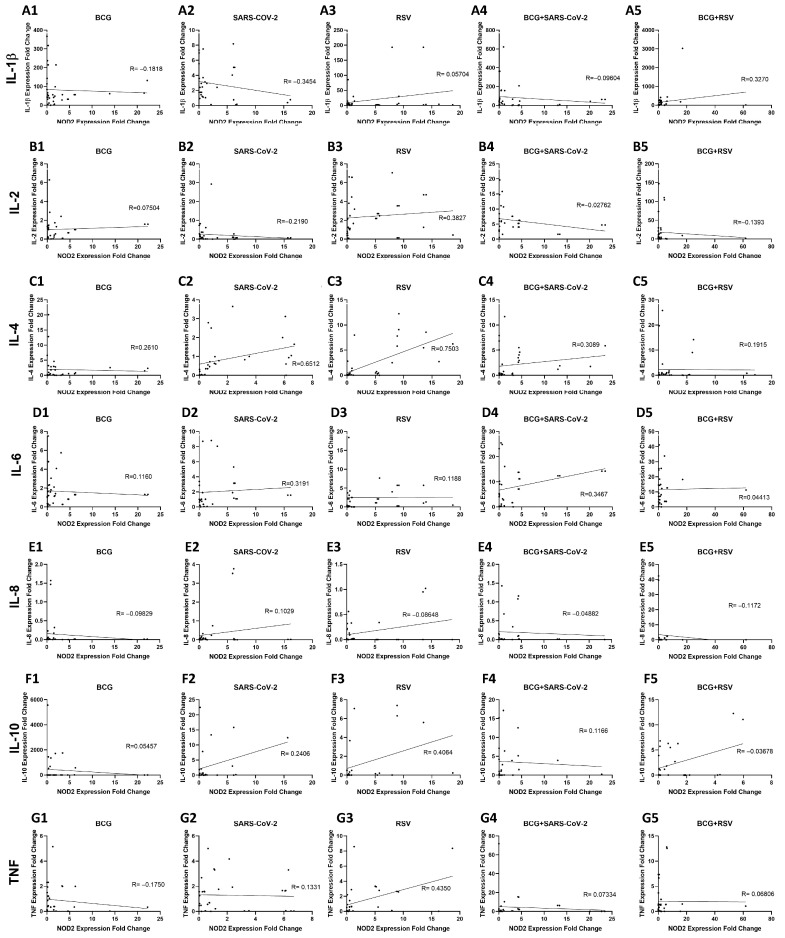
Correlation between NOD2 receptor expression and cytokine expression in whole-blood cultures stimulated with BCG and SARS-CoV-2 and RSV virus antigens; RSV(+), group seropositive for RSV infection; ((**A**): IL-1β; (**A1**)-BCG, (**A2**)-SARS-CoV-2; (**A3**)-RSV; (**A4**)-BCG+SARS-CoV-2; (**A5**)-BCG+RSV. (**B**): IL-2; (**B1**)-BCG, (**B2**)-SARS-CoV-2; (**B3**)-RSV; (**B4**)-BCG+SARS-CoV-2; (**B5**)-BCG+RSV. (**C**): IL-4; (**C1**)-BCG, (**C2**)-SARS-CoV-2; (**C3**)-RSV; (**C4**)-BCG+SARS-CoV-2; (**C5**)-BCG+RSV. (**D**): IL-6; (**D1**)-BCG, (**D2**)-SARS-CoV-2; (**D3**)-RSV; (**D4**)-BCG+SARS-CoV-2; (**D5**)-BCG+RSV. (**E**): IL-8; (**E1**)-BCG, (**E2**)-SARS-CoV-2; (**E3**)-RSV; (**E4**)-BCG+SARS-CoV-2; (**E5**)-BCG+RSV. (**F**): IL-10; (**F1**)-BCG, (**F2**)-SARS-CoV-2; (**F3**)-RSV; (**F4**)-BCG+SARS-CoV-2; (**F5**)-BCG+RSV. (**G**): TNF; (**G1**)-BCG, (**G2**)-SARS-CoV-2; (**G3**)-RSV; (**G4**)-BCG+SARS-CoV-2; (**G5**)-BCG+RSV). SARS-CoV-2(+), group seropositive for SARS-CoV-2; RSV(+)SARS-CoV-2(+), group seropositive for RSV and SARS-CoV-2; RSV(−)SARS-CoV-2(−), group seronegative for RSV and SARS-CoV-2, BCG, bacillus Calmette-Guérin, SARS-CoV-2, severe acute respiratory syndrome coronavirus 2; RSV, respiratory syncytial virus. Differences in the levels of CD14, HLA-DR, CD11b, and CD206 concentrations between the studied groups were compared using the Spearman correlation. A *p*-value was considered significant if <0.05.

As illustrated in Figure 9(A1–A5), a range of correlation plots are provided, which demonstrate the relationship between NOD2 and IL-1β mRNA expression levels under various stimulation conditions. In whole blood cultures stimulated with SARS-CoV-2 (Figure 9(A2)), a moderate negative correlation was observed (R = −0.3454, *p* = 0.0285). Conversely, in those co-stimulated with BCG and RSV antigens (Figure 9(A5)), a moderate positive correlation was detected (R = 0.3270, *p* = 0.0241). In turn, Figure 9(B1–B5) illustrate the correlation between NOD2 and IL-2 mRNA expression levels. In RSV-stimulated cultures (Figure 9(B3)), a moderate positive correlation (R = 0.3827, *p* = 0.0097) was observed. In the other groups, there were no correlations. The relationship between NOD2 and IL-4 mRNA is shown in Figure 9(C1–C5). A strong positive correlation was observed in SARS-CoV-2-stimulated cultures (Figure 9(C2), R = 0.6512, *p* = 0.0001). Similarly, under RSV stimulation (Figure 9(C3)), a very strong positive correlation was detected (R = 0.7503, *p* < 0.0001). In those co-stimulated with BCG+SARS-CoV-2 (Figure 9(C4)), a moderate positive correlation was found (R = 0.3089, *p* = 0.0378). In contrast, in BCG-only (Figure 9(C1)) and BCG+RSV-stimulated cultures (Figure 9(C5)), there were no correlations. The correlation between NOD2 and IL-6 mRNA expression is presented in Figure 9(D1–D5). A moderate positive correlation (R = 0.3191, *p* = 0.0375) was identified in the SARS-CoV-2-antigen-stimulated cultures (Figure 9(D2)). Similarly, a moderate correlation (R = 0.3467, *p* = 0.0413) was observed in the BCG + SARS-CoV-2-co-stimulated group (Figure 9(D4)). In the other groups, no correlation was observed between mRNA expression levels of NOD2 and IL-6. Figure 9(E1–E5) show the correlation between NOD2 mRNA and IL-10 expression. No correlation between NOD2 mRNA and IL-10 was observed in any culture. In turn, Figure 9(F1–F5) show the relationships between NOD2 mRNA levels and IL-8. A positive correlation (R = 0.4064, *p* < 0.05) was observed in whole-blood cultures stimulated with RSV antigens only (Figure 9(F5)), while in the other cases, no correlation was observed between NOD2 mRNA levels and IL-8. Figure 9(F1–F5) show the correlations between *NOD2* and *IL-8* mRNA expression. Finally, Figure 9(G1–G5) illustrate the correlation between *NOD2* and *TNF* mRNA expression under different stimulation conditions. A positive correlation (R = 0.4350, *p* = 0.0081) was solely observed within the context of the RSV-antigen-stimulated culture (Figure 9(G3)).

### 3.11. Expression Levels of CD14, HLA-DR, CD11b, and CD206 on Monocytes

**Figure 10:**
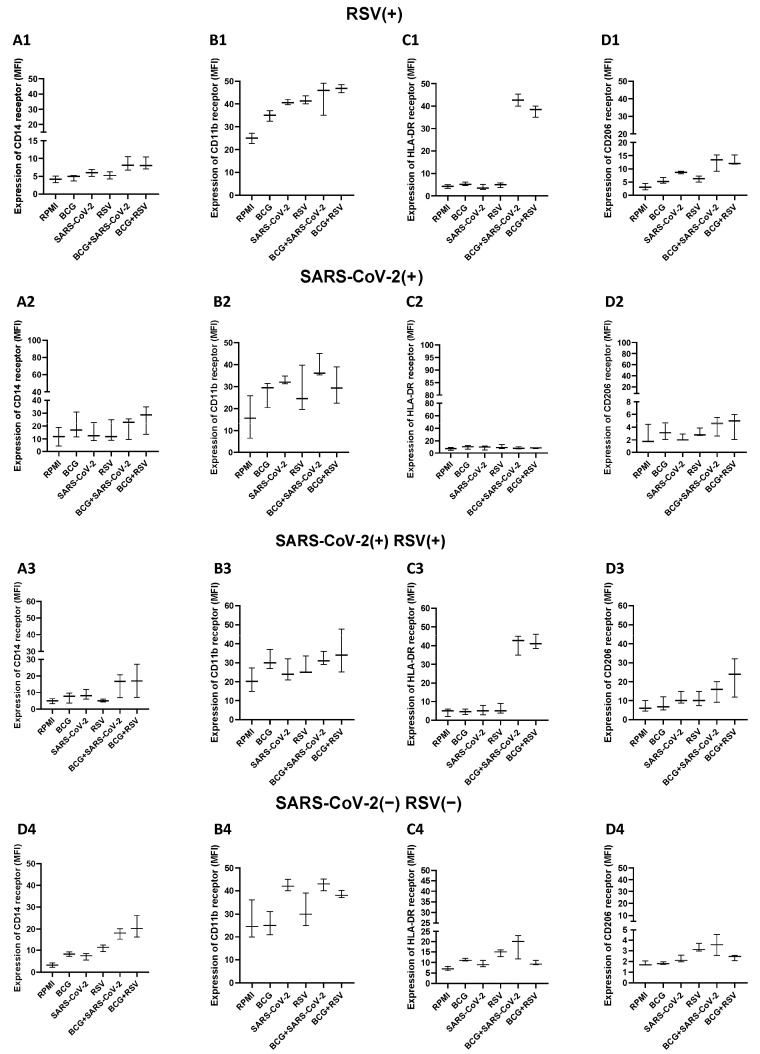
Density levels of CD14 (panel (**A1**–**A4**)), HLA-DR (panel (**B1**–**B4**)), CD11b (panel (**C1**–**C4**)), and CD206 (panel (**D1**–**D4**)) receptors on monocytes after 48 h stimulation of diluted whole-blood samples from BCG-vaccinated children; RSV(+), group seropositive for RSV infection; SARS-CoV-2(+), group seropositive for SARS-CoV-2; RSV(+)SARS-CoV-2(+), group seropositive for RSV and SARS-CoV-2; RSV(−)SARS-CoV-2(−), group seronegative for RSV and SARS-CoV-2, BCG, bacillus Calmette-Guérin, SARS-CoV-2, severe acute respiratory syndrome coronavirus 2; RSV, respiratory syncytial virus. Differences in the levels of CD14, HLA-DR, CD11b, and CD206 concentrations between the studied groups were compared using the non-parametric Kruskal-Wallis Two-way ANOVA with Dunn’s post-test. A *p*-value was considered significant if <0.05.

To assess monocyte activation and its association with altered cytokine and chemokine responses to non-specific stimuli in BCG-vaccinated children, we performed flow cytometric analysis of peripheral blood leukocytes (PBLs) isolated from 48 h diluted whole-blood stimulation assays. Cells were stimulated with RSV or SARS-CoV-2 antigens and subsequently stained for surface markers indicative of the monocyte phenotype and activation status. In line with the BCG-induced enhancement of broad cytokine and chemokine responses upon stimulation with heterologous antigens, we observed upregulated expression of CD14 across all child cohorts (Figure 10). Furthermore, increased HLA-DR expression was noted in monocytes from children seropositive for RSV (RSV(+)) and those co-exposed to both RSV and SARS-CoV-2 (RSV(+)SARS-CoV-2(+)), suggesting an elevated state of monocyte activation in the context of prior viral exposure and BCG priming. Interestingly, no significant BCG-related modulation was detected in the expression of CD11b across any of the study groups in response to these non-specific stimuli (Figure 10). In contrast, although overall group differences in CD206 expression did not reach statistical significance, a trend toward increased CD206 expression was evident in the RSV(+)SARS-CoV-2(+) group. This may reflect a more nuanced, stimulus-dependent modulation of monocyte function, possibly involving alternative activation pathways.

## 4. Discussion

In this study, we investigated how BCG vaccination modulates immune responses in children with varying serostatuses for SARS-CoV-2 and RSV, using an in vitro whole-blood stimulation model. We assessed the mRNA expression of key cytokines (IL-2, IL-6, IL-1β, IL-8, TNF, IL-4, IL-10), as well as the pattern recognition receptor NOD2, following stimulation with viral antigens, either alone or in combination with live BCG. Our findings emphasize the complex and dynamic nature of BCG-induced immunomodulation, demonstrating that prior viral exposure markedly influences cytokine profiles. This effect appears to be at least partly mediated through NOD2-dependent pathways, suggesting that BCG may reprogram innate immune responses in a context-specific manner.

The BCG vaccine, traditionally used against tuberculosis, has shown potential benefits in protecting against various infections through broad activation of the immune system [1]. The ability of BCG to enhance non-specific immunity may be crucial in the control of respiratory pathogens [18]. In agreement with previous studies [3,7], we observed that co-stimulation with BCG and RSV antigens led to the significant upregulation of NOD2 mRNA expression, particularly in children who were RSV-seropositive and SARS-CoV-2-seronegative/RSV-seronegative. Interestingly, we found that children with prior SARS-CoV-2 exposure (SARS-CoV-2 seropositive) exhibited the highest NOD2 expression following RSV antigen stimulation alone. This suggests that previous SARS-CoV-2 infection may alter NOD2 responsiveness to subsequent microbial stimuli, potentially shaping the immune system’s ability to respond to new infections. This observation is in line with the study by [19], which demonstrated that aerosol-delivered BCG vaccination in rhesus macaques resulted in increased cytokine responses following SARS-CoV-2 challenge. Moreover, these findings corroborate reports from [20], who described altered cytokine profiles in individuals with severe COVID-19, further supporting the notion that prior viral infections may modulate immune system responses.

Cytokine expression patterns were markedly influenced by BCG vaccination. Our study showed the differential expression of key pro-inflammatory cytokines (IL-1β, IL-6, TNF) depending on the individuals’ serological status and BCG stimulation, suggesting an important role for this vaccine in shaping the immune response against viral pathogens. Notably, we observed a significant increase in IL-1β and IL-6 mRNA levels in SARS-CoV-2-seropositive children following co-stimulation with BCG and SARS-CoV-2 antigens, suggesting that BCG vaccination enhances innate immune responses to SARS-CoV-2. These findings are in agreement with those of [19], who showed the similar upregulation of pro-inflammatory cytokines in rhesus macaques vaccinated with BCG before SARS-CoV-2 exposure. IL-6, in particular, was upregulated across all groups, reflecting its dual role in both pro-inflammatory and anti-inflammatory responses [21]. This observation corresponds with earlier studies indicating that IL-6 plays a critical role in both protection and pathology during respiratory viral infections [20,22]. IL-6 is rapidly produced in the lungs and airways following RSV infection, with its levels often remaining elevated throughout the course of the disease [22,23] (McNamara et al., 2003; Levitz et al., 2012). As a key regulator of the inflammatory response, IL-6 promotes the maturation of regulatory T cells (Tregs) and limits excessive Th1 activity [24]. Through these mechanisms, IL-6 may reduce the severity of RSV-induced pathology while maintaining effective viral clearance. Previous studies have demonstrated that the early depletion of IL-6 during infection leads to an increased viral load, more severe inflammatory responses, and greater lung damage [25]. Furthermore, Besteman et al. (2022) examined the impact of IL-6 on RSV infection and found that CD14-deficient patients showed reduced IL-6 expression, resulting in the inadequate control of RSV infection [26]. In our earlier research, we showed that the stimulation of cells with BCG antigens can affect the interferon response to RSV antigens, revealing significant differences in the secretion and mRNA expression of IFN-α and IFN-γ in response to both RSV antigens and the BCG vaccine [27]. Our current results build upon these studies, demonstrating the effect of BCG stimulation on additional cytokines. Specifically, we observed increased IL-6 expression in cell cultures co-stimulated with BCG and RSV. This effect was especially pronounced in seronegative patients. In the SARS-CoV-2(−)RSV(−) group, BCG stimulation, in the absence of prior viral exposure, modulated the pro-inflammatory response without attenuating it. Elevated IL-6 expression in these cases may aid in controlling RSV infection by enhancing regulatory mechanisms within the inflammatory response. The elevated IL-2 levels in BCG+SARS-CoV-2-co-stimulated cultures further corroborate the ability of BCG to induce robust immune responses, as seen in other studies on trained immunity [28]. A particularly noteworthy observation was the significant increase in IL-10 expression, especially in cultures co-stimulated with BCG and SARS-CoV-2. IL-10 is a well-known regulatory cytokine that plays a crucial role in limiting excessive inflammation while promoting antiviral responses [29]. This suggests that stimulation with the BCG vaccine not only boosts immune activation but also helps regulate the immune response to prevent immunopathology, particularly during viral infections. This observation is consistent with findings from [19] and studies on the role of IL-10 in mitigating inflammation during viral infections.

An imbalance between Th1 and Th2 cells is thought to be responsible for the severity of disease in RSV-infected individuals [30]. A dominant Th2 response marked by elevated levels of IL-4, IL-5, IL-6, and IL-10 has been associated with ineffective viral clearance and more severe disease symptoms. In turn, Th1 cells, which produce IFN-γ, TNF, and IL-2, promote the activity of cytotoxic T cells and NK cells, enabling effective viral clearance [31,32]. It is known that a severe course of RSV infection is associated with an excessive Th2 (IL-4) and a reduced Th1 (IFN-γ) response [33,34]. In earlier studies, we showed that the stimulation of whole-blood cells with the BCG vaccine can modulate the interferon (IFN-γ) response during viral infections [27]. However, our current data suggest a more complex effect of BCG stimulation on immunity against RSV, involving both protective (Th1) and potentially damaging (Th2) pathways. We observed that co-stimulation with BCG and RSV antigens led to increased mRNA expression of both cytokines associated with Th1-type responses (e.g., IL-2) and cytokines specific to Th2-type responses (e.g., IL-4, IL-10, and IL-6). Elevated levels of IL-2 and IL-4 may suggest that the BCG vaccine promotes the simultaneous activation of Th1 and Th2 responses, which would control inflammation while eliminating the virus. However, it must be borne in mind that simultaneous increases in both cytokines do not always imply a perfect balance between these types of responses. Interestingly, the observed simultaneous increase in IL-2 and IL-4 following combined BCG + RSV stimulation suggests an integrated, rather than polarized, T helper response. IL-2 is central to Th1-driven T cell proliferation and cytotoxic function, while IL-4 promotes Th2 differentiation and humoral immunity. The concurrent upregulation of both cytokines may represent an intermediate or transitional state, enabling a balanced immune response that leverages Th1-mediated cellular defenses (crucial for intracellular pathogens like RSV) alongside Th2 mechanisms that modulate inflammation or prime the system for future antigen exposure. This pattern corroborates findings from other contexts, where high doses of BCG have induced mixed Th1/Th2 responses in mice (e.g., lower doses favor a Th1 bias, but higher doses shifted toward mixed responses) [35]. Moreover, heterologous priming with BCG has been shown to produce the context-dependent activation of both innate and adaptive immunity, supporting trained immunity and broad immunological adaptability. Notably, studies of recombinant BCG-based RSV vaccines in humans indicate that such platforms can elicit robust IFN-γ and IL-2 secretion alongside a measured IL-4 release, reflecting similarly balanced immune activation. Thus, our data align with mounting evidence that BCG does not strictly skew immunity toward Th1 responses but can facilitate versatile and heterogeneous cytokine milieus, tailored to the pathogen context. Future work should dissect the T cell subset dynamics to clarify the downstream effects of this mixed cytokine profile.

Interestingly, we observed stronger IL-2 and IL-6 mRNA expression in the seronegative groups (SARS-CoV-2(−)/RSV(−)) after stimulation with BCG and RSV antigens. The seronegativity to these viruses indicates that the immune system of these individuals had no previous contact with these viruses. Consequently, their immune cells may be more reactive to new antigens, resulting in stronger cytokine mRNA expression. This finding aligns with the concept of “trained immunity” [36], in which prior exposure to one pathogen, such as through BCG vaccination, can enhance the immune system’s response to novel pathogens.

Our study suggests the central role of NOD2 in shaping immune responses to BCG and viral antigens. We observed that NOD2 expression was positively correlated with the production of several cytokines, including IL-1β, IL-2, IL-4, IL-6, IL-10, and TNF (Figures S1–S10), indicating that NOD2 may act as a key upstream regulator of cytokine responses [4]. NOD2 has previously been implicated in the modulation of both innate and adaptive immune responses to infections [3,7]. In this context, our data suggest that the NOD2-dependent regulation of cytokine production plays a pivotal role in the heterologous immunity induced by BCG stimulation. Interestingly, our results indicate that NOD2 may have a distinct role depending on the serological status of the individuals. In the SARS-CoV-2-seropositive group, NOD2 expression correlated positively with IL-4 and IL-6 after SARS-CoV-2 stimulation, indicating that NOD2 may influence both inflammatory and regulatory pathways in previously infected individuals. Conversely, in the seronegative children, we observed an inverse correlation between NOD2 and TNF, suggesting a potential regulatory role for NOD2 in preventing excessive inflammation in naïve immune systems. These observations are in line with previous research, which showed altered NOD2 expression in response to COVID-19 severity [37], although our study did not detect significant differences in NOD2 mRNA expression in SARS-CoV-2-stimulated cultures. Interestingly, our results indicate that NOD2 may have a distinct role depending on the serological status of the individuals. For example, in the SARS-CoV-2(+) group, NOD2 expression correlated positively with IL-4 and IL-6 after SARS-CoV-2 stimulation, suggesting that NOD2 may influence both inflammatory and regulatory pathways in previously infected hosts. Conversely, in seronegative children, we observed an inverse correlation between NOD2 and TNF, which may indicate a regulatory role for NOD2 in preventing excessive inflammation in naïve immune systems. This aligns with findings by [37], who observed altered NOD2 expression in response to COVID-19 severity, although our study did not detect significant differences in NOD2 mRNA expression in SARS-CoV-2-stimulated cultures. The role of NOD2 in immune regulation was further supported by our observation that NOD2 expression was correlated with IL-10 levels, particularly in cultures co-stimulated with BCG and RSV antigens. This suggests that NOD2 may help balance the immune response, promoting anti-inflammatory cytokine production to limit immunopathology during viral infections. This is particularly relevant as excessive inflammation, as observed in diseases like COVID-19 [20], can lead to severe disease outcomes.

The findings also suggest that prior viral exposure, whether from RSV or SARS-CoV-2, significantly influences immune responses to BCG antigen stimulation. For instance, in the RSV-seropositive group, we observed elevated IL-1β and IL-10 mRNA levels following co-stimulation with BCG and RSV, which mirrors earlier findings by [38], showing enhanced cytokine production following RSV infection. These results imply that viral exposure may “prepare” the immune system, priming it for a more robust inflammatory response upon subsequent stimulation, a concept that aligns with the idea of immune memory and trained immunity [3]. Interestingly, we observed that seronegative children (SARS-CoV-2(−)/RSV(−)) showed stronger IL-2 and IL-6 mRNA responses to BCG and viral antigen co-stimulation. This suggests that the immune system of seronegative individuals, without prior viral exposure, may respond more vigorously to new antigens, reflecting the enhanced capacity of trained immunity to prime naïve immune systems [36]. These findings also complement our previous observations of differential interferon responses to RSV antigens in BCG-vaccinated individuals [27].

The differential cytokine profiles observed in this study, which varied with prior viral exposure and antigenic stimulation in BCG-vaccinated children, highlight the complex interactions between past infections and immune responses to respiratory pathogens. The ability of BCG to induce both pro-inflammatory and regulatory cytokines highlights its complex role in immune regulation. This dual effect may provide a mechanism to boost antiviral defenses while minimizing the risk of immunopathology, a critical factor in managing diseases like COVID-19. Furthermore, the insights gained from this study pave the way for designing novel immunomodulatory strategies to enhance protection against respiratory infections, especially in the context of emerging pathogens such as SARS-CoV-2.

In our study, we did not detect any significant differences in the polymorphism of the NOD2 gene, which may limit the interpretation of the differential patterns of immune responses observed in children vaccinated with BCG. Similarly, the absence of mutations in the NOD2/CARD15 gene was also reported in a study by Yamazaki et al. among 483 Japanese patients with Crohn’s disease [39]. Thus, the mechanism of impaired immunity associated with the 3020insC mutation is unlikely to play an important role in the immune response of children in this age group in Poland. The potential influence of genetic variations in NOD2 on immune responses, particularly in relation to BCG-induced immune modulation, has been well-documented in previous studies. For instance, research by [40] emphasizes that genetic variations in NOD2 may influence both innate and adaptive immune responses to stimuli such as BCG, suggesting a potential interaction between genetic factors and vaccine-induced immune training. The study demonstrated that γ-irradiated BCG had long-term effects on immune responses, with the significant modulation of cytokine profiles in both in vitro and in vivo models. However, this modulation was not solely attributed to NOD2 polymorphisms, suggesting that other factors, including epigenetic changes and broader immune system reprogramming, may also play crucial roles in BCG’s effectiveness. The findings from Arts et al. [40] further highlight the intricate and multifaceted nature of BCG-induced immunity. They found that BCG vaccination can have lasting effects on the immune system, extending well beyond the direct response to *Mycobacterium tuberculosis*. Their work demonstrated that the immune system’s responsiveness to secondary pathogens, such as viruses, was enhanced after BCG vaccination, a phenomenon attributed to the induction of trained immunity [40]. This concept, which involves the long-term reprogramming of innate immune cells to respond more robustly to subsequent infections, aligns with our observation that BCG stimulation affects cytokine expression in children with varying serostatuses to SARS-CoV-2 and RSV. Although our study did not observe significant differences in NOD2 gene polymorphisms in BCG-vaccinated children, it cannot be excluded that subtle genetic differences may shape patterns of the immune response to BCG stimulation and viral stimuli. It is worth noting that NOD2 is a key player in innate immunity, particularly in the recognition of bacterial components and the activation of downstream signaling pathways, such as the NF-κB pathway, which modulates the production of pro-inflammatory cytokines. Given the central role of NOD2 in maintaining immune homeostasis and its involvement in recognizing pathogens like RSV, as well as modulating responses to BCG vaccination, the absence of genetic differences in our study suggests that other factors may be more prominent in shaping the immune response. Future research focused on exploring the impact of NOD2 polymorphisms in more detail, particularly in relation to trained immunity and vaccine responses, would be valuable in further understanding the genetic underpinnings of immune responses to BCG and its potential role in mitigating respiratory infections like RSV and SARS-CoV-2.

The present data confirm that BCG-primed children mount a quantitatively and qualitatively altered monocyte response when confronted with heterologous viral antigens. The increased expression of CD14 and HLA-DR observed in children after RSV or SARS-CoV-2 infections may be indicative of prolonged monocyte activation and persistent changes in the non-specific response, dependent on previous exposure to pathogens. The similar upregulation of CD14, TLR4, and CD11b was first described three months after BCG vaccination in adults, where it was shown to depend on the NOD2-driven epigenetic remodeling of monocytes. More recently, Ahmed et al. demonstrated that BCG revaccination boosts pro-inflammatory cytokine production by HLA-DR^+^CD14+ CD16^−^ cells in adults, further corroborating the link between BCG and sustained monocyte activation. Our findings extend those observations to the pediatric setting and indicate that prior infection by common respiratory viruses provides an additional layer of stimulus-specific imprinting [41]. Interestingly, we did not observe the significant upregulation of CD11b on monocytes following BCG or RSV stimulation, despite clear evidence of activation through increased cytokine production and elevated expression of CD14 and HLA-DR. This finding contrasts earlier studies in adults, where CD11b upregulation was reported following BCG vaccination. Several factors may explain this discrepancy. Firstly, age-dependent kinetics are likely to play a role. Our study focused on pediatric participants whose samples were collected relatively soon after BCG vaccination in newborns, whereas studies involving adults typically included longer intervals between vaccination and analysis. It is possible that CD11b expression peaks at different times in infants and may have been missed in our sampling window. It is also known that CD11b undergoes rapid internalization upon activation, particularly in response to viral ligands. This internalization may reduce surface detectability via flow cytometry, even if the receptor is functionally active. Additionally, the heterogeneity of monocyte subsets and the nature of the stimulus (e.g., live virus vs. vaccine) may influence the degree and timing of CD11b expression. Context-dependent differences in marker expression have been observed in other models. For example, in macaque infants, BCG administered to newborns selectively enhanced cytokine-rich responses without causing a uniform increase in all canonical activation markers, including CD11b. This suggests that BCG and RSV may activate monocytes through pathways that do not strongly induce CD11b or that regulatory feedback mechanisms limit its surface expression to prevent excessive inflammation [42]. Future studies incorporating kinetic analyses and subset-specific phenotyping will be valuable to clarify the role of CD11b in monocyte activation under these conditions.

The tendency toward higher CD206 (mannose receptor) expression in the RSV(+)SARS-CoV-2(+) subgroup adds nuance to this picture. RSV infection of a three-dimensional human lung model was recently shown to drive the differentiation of blood monocytes toward a CD206+ HLA-DR+ phenotype that retains antiviral functionality while acquiring tissue-resident features [43]. Considering that BCG is capable of priming both classical and alternative activation pathways, the modest CD206 upregulation observed may indicate a transition toward a reparative or immunoregulatory state, apparent only in children who have undergone sequential viral exposures.

Taken together, our results support a model in which BCG-induced innate immunity does not merely amplify baseline immune signaling but reprograms monocytes to respond in a stimulus- and history-dependent manner. The upregulation of CD14 and HLA-DR reflects a shared activation phenotype, while the differential expression of CD11b and CD206 suggests more nuanced modulation shaped by the timing of BCG exposure and the nature of subsequent viral encounters. These receptor-level adaptations likely act in concert with epigenetically imprinted transcriptional changes, enabling a broad yet highly calibrated immune response. Such reprogramming may underlie the heterologous protection observed after BCG vaccination against unrelated respiratory pathogens, including RSV and SARS-CoV-2 [41].

This study has several important limitations that should be considered when interpreting the results. First, all participants were BCG-vaccinated children, and we did not include a BCG-unvaccinated control group due to national vaccination policies and ethical constraints. Therefore, it is not possible to definitively attribute the observed immune response patterns solely to BCG vaccination. Second, multiple cytokines were tested simultaneously, which increases the risk of false positive results. We addressed this by applying the Benjamini-Hochberg correction to control the false discovery rate. However, this approach may also increase the chance of false negatives, which should be considered in the interpretation of non-significant findings. Third, as with any observational study, potential confounding variables such as environmental exposures, the genetic background, and health status could influence immune responses. Although we controlled for major confounders where possible, unmeasured factors may have contributed to the observed variability. Finally, an important limitation of this study is the relatively small and uneven sample size across the four groups, which may reduce the statistical power. The analysis indicated that with our sample size (N = 48) and assuming a medium effect size (f = 0.25), the achieved power was 69%. This increases the risk of type II errors, particularly for smaller subgroups, and may limit the detection of biologically relevant effects. While we applied non-parametric methods and implemented several analytical controls, we recognize that these findings should be interpreted with caution. This study is exploratory in nature, and the results should be validated in larger, prospectively powered cohorts. Nevertheless, the effect size estimates generated here may serve as a basis for sample size planning in future studies.

## 5. Conclusions

In summary, our results indicate that in BCG-vaccinated children, immune responses to respiratory viruses are associated with differential NOD2-dependent cytokine expression, which may reflect the influence of both prior viral exposures and antigenic stimulation. The induction of both pro-inflammatory and regulatory cytokines in response to RSV and SARS-CoV-2 antigens in BCG-vaccinated children suggests a balanced immune profile, potentially shaped by prior antigenic exposures and immune training. These results underscore the therapeutic potential of BCG in strengthening host immunity against respiratory infections and offer valuable insights for the development of future vaccine strategies and immunomodulatory interventions.

## Figures and Tables

**Table 1:** The characteristics of the groups in the study.

Parameter	Total	Groups			
		RSV(+)	SARS-CoV-2(+)	RSV(+) SARS- CoV-2(+)	RSV(−) SARS- CoV-2(−)
Ethnicity	Caucasia	Caucasian	Caucasian	Caucasian	Caucasian
N	48	14	17	11	6
Sex					
Girls, *n* (%)	23 (48%)	8 (57%)	5 (30%)	6 (55%)	4 (66%)
Boys, *n* (%)	25 (52%)	6 (43%)	12 (70%)	5 (45%)	2 (33%)
Age median	8	9	9	8	8
Age range	6–12	7–12	6–12	7–12	7–12
BCG vaccination	100%	100%	100%	100%	100%
Compliance with the Polish vaccination schedule	Yes	Yes	Yes	Yes	Yes
General health status	Good	Good	Good	Good	Good
Chronic respiratory diseases	No	No	No	No	No
Form of immunodeficiency	No	No	No	No	No
Acute infection <30 days prior to blood collection	No	No	No	No	No
Antibiotics <30 days prior to blood collection	No	No	No	No	No
Hospitalized for severe RSV or SARS-CoV-2	No	No	No	No	No

Abbreviations: RSV—respiratory syncytial virus; SARS-CoV-2—severe acute respiratory syndrome coronavirus 2. The differences between the groups studied did not reach statistical significance with regard to age (ANOVA with Dunn’s post-test test, *p* > 0.05) and sex (Chi-square test or Fisher’s exact test, *p* > 0.05).

**Table 2:** Starters and temperatures of annealing selected for expression analysis.

	Sequence	Temperature of Annealing	Source [Reference]
R702W	forward 5′-AGATCACAGCAGCCTTCCTG-3′, reverse 5′-CACGCTCTCTTGGCCTCACC-3′	62	[14]
G908R	forward 5′-CTCTTGGCCTTTCAGATTCTG-3′ reverse 5′-CAGCTCCTCCCTCTTCACCT-3	54	[14]
1007fs	forward 5′-GGCAGAAGCCCTCCTGCAGGCC-3′ reverse 5′-CCTCAAAATTCTGCCATTCC-3′	60	[14]
IL-2	forward 5′-AGAATCCCAAACTCACCAGGA-3′ reverse 5′-TGCTGATTAAGTCCCTGGGT-3′	60	[16]
IL-4	forward 5′-GCAGTTCTACAGCCACCATG-3′ reverse 5′-ACTCTGGTTGGCTTCCTTCA-3′	60	[16]
IL-6	forward 5′-CCTTCCAAAGATGGCTGAAA-3′ reverse 5′-CAGGGGTGGTTATTGCATCT-3′	60	[16]
IL-1B	qHsaCID0022272	60	Bio-Rad
IL-8	qHsaCED0046633	60	Bio-Rad
TNF	qHsaCED0037461	60	Bio-Rad
IL-10	QHsaCED0044704	60	Bio-Rad

**Table 3:** Genotype distribution of the G908R, R702W, and 1007fs polymorphisms in the NOD2 gene.

NOD2	Genotyping	N (%)
G908R	G/G	40 (100)
	R/R	0 (0)
	G/R	0 (0)
R702W	R/R	40 (100)
	W/W	0 (0)
	R/W	0 (0)
1007fs	Leu/Leu	40 (100)
	Pro/Pro	0 (0)
	Leu/Pro	0 (0)

## Data Availability

The original contributions presented in this study are included in the article. Further inquiries can be directed to the corre-sponding author(s).

## Supplementary Materials

The following supporting information can be downloaded at: https://www.mdpi.com/article/10.3390/pathogens14070683/s1, Figure S1: Genotyping 1007fs; Figure S2: Genotyping G908R; Figure S3: Genotyping R702W; Figure S4: Interdependence of NOD2 and IL-1β expression in whole-blood cultures following stimulation with BCG, SARS-CoV-2, and RSV antigens; Figure S5: Interdependence of NOD2 and IL-2 expression in whole-blood cultures following stimulation with BCG, SARS-CoV-2, and RSV antigens; Figure S6: Interdependence of NOD2 and IL-4 expression in whole-blood cultures following stimulation with BCG, SARS-CoV-2, and RSV antigens; Figure S7: Interdependence of NOD2 and IL-6 expression in whole-blood cultures following stimulation with BCG, SARS-CoV-2, and RSV antigens; Figure S8: Interdependence of NOD2 and IL-8 expression in whole-blood cultures following stimulation with BCG, SARS-CoV-2, and RSV antigens; Figure S9: Interdependence of NOD2 and IL-10 expression in whole-blood cultures following stimulation with BCG, SARS-CoV-2, and RSV antigens; Figure S10: Interdependence of NOD2 and TNF expression in whole-blood cultures following stimulation with BCG, SARS-CoV-2, and RSV antigens.

## Abbreviations

The following abbreviations are used in this manuscript:

BCG	Bacillus Calmette-Guérin
SARS-CoV-2	Directory of open access journals
RSV	Three letter acronym
NOD2	Nucleotide-binding oligomerization domain-containing protein 2
H3K4me3	Histone 3 at lysine 4
IL	Interleukin
HPV	Human papillomavirus
HSV	Herpes simplex virus
Th1	Type 1 helper lymphocyte
IFN	Interferon
rBCG-RSV	Recombinant BCG vaccine expressing the RSV nucleoprotein
N	number
