# Supplementary material for: NOD2 (Nucleotide-Binding Oligomerization Domain-Containing Protein 2)-Mediated Modulation of the Immune Response Induced by BCG (Bacillus Calmette-Guérin) Bacilli
Source: Pathogens. 2025 Jul 11;14(7):683. doi: 10.3390/pathogens14070683 (PMC12300030; doi:10.3390/pathogens14070683)
Supplement: Supplementary file 1 [file pathogens-14-00683-s001.zip › Fig.S1-S3.docx]

 
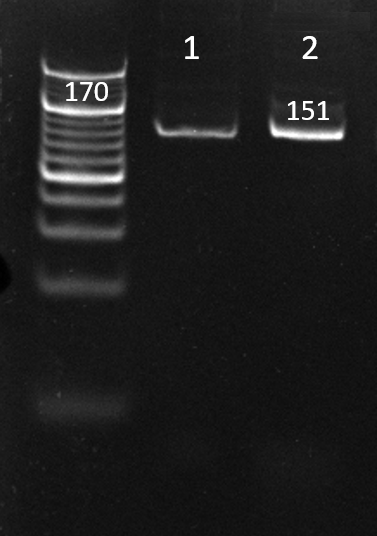


**Fig. 1S** Genotyping 1007fs; 1-PCR product, 2- PCR product after digestion Leu1007Leu homozygotes (151bp).

1 2

 
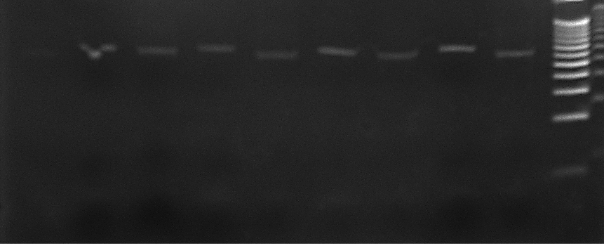


170

163

**Fig. 2S** Genotyping G908R; 1-PCR product, 2- PCR product after digestion G908G homozygotes (163bp).


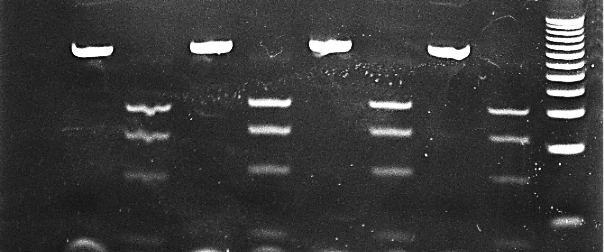

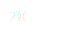


1 2

75

50

25

76

54

35

20

**Fig. 3S** Genotyping R702W; 1-PCR product, 2- PCR product after digestion R702R homozygotes (20, 35, 54, 76 bp).
