# Supplementary material for: NOD2 (Nucleotide-Binding Oligomerization Domain-Containing Protein 2)-Mediated Modulation of the Immune Response Induced by BCG (Bacillus Calmette-Guérin) Bacilli
Source: Pathogens. 2025 Jul 11;14(7):683. doi: 10.3390/pathogens14070683 (PMC12300030; doi:10.3390/pathogens14070683)
Supplement: Supplementary file 1 [file pathogens-14-00683-s001.zip › Fig.S4-S10.docx]

**Fig. S4** Correlation between NOD2 receptor expression and IL-1β expression in whole blood cultures stimulated with BCG and SARS-CoV-2 and RSV virus antigens**. Abbreviations**; RSV(+), group seropositive for RSV infection; SARS-CoV-2(+), group seropositive for SARS-CoV-2; RSV(+)SARS-CoV-2(+), group seropositive for RSV and SARS-CoV-2; RSV(-)SARS-CoV-2(-), group seronegative for RSV and SARS-CoV-2, BCG, bacillus Calmette–Guérin, SARS-CoV-2, severe acute respiratory syndrome coronavirus 2; RSV, respiratory syncytial virus. The Spearman correlation test was used to assess the correlation of NOD2 mRNA expression and IL-8 in the different study groups. A p-value was considered significant if < 0.05.

**Fig. S5** Correlation between NOD2 receptor expression and IL-2 expression in whole blood cultures stimulated with BCG and SARS-CoV-2 and RSV virus antigens**. Abbreviations**; RSV(+), group seropositive for RSV infection; SARS-CoV-2(+), group seropositive for SARS-CoV-2; RSV(+)SARS-CoV-2(+), group seropositive for RSV and SARS-CoV-2; RSV(-)SARS-CoV-2(-), group seronegative for RSV and SARS-CoV-2, BCG, bacillus Calmette–Guérin, SARS-CoV-2, severe acute respiratory syndrome coronavirus 2; RSV, respiratory syncytial virus. The Spearman correlation test was used to assess the correlation of NOD2 mRNA expression and IL-8 in the different study groups. A p-value was considered significant if < 0.05.

**Fig. S6** Correlation between NOD2 receptor expression and IL-4 expression in whole blood cultures stimulated with BCG and SARS-CoV-2 and RSV virus antigens**. Abbreviations**; RSV(+), group seropositive for RSV infection; SARS-CoV-2(+), group seropositive for SARS-CoV-2; RSV(+)SARS-CoV-2(+), group seropositive for RSV and SARS-CoV-2; RSV(-)SARS-CoV-2(-), group seronegative for RSV and SARS-CoV-2, BCG, bacillus Calmette–Guérin, SARS-CoV-2, severe acute respiratory syndrome coronavirus 2; RSV, respiratory syncytial virus. The Spearman correlation test was used to assess the correlation of NOD2 mRNA expression and IL-8 in the different study groups. A p-value was considered significant if < 0.05.

**Fig. S7** Correlation between NOD2 receptor expression and IL-6 expression in whole blood cultures stimulated with BCG and SARS-CoV-2 and RSV virus antigens**. Abbreviations**; RSV(+), group seropositive for RSV infection; SARS-CoV-2(+), group seropositive for SARS-CoV-2; RSV(+)SARS-CoV-2(+), group seropositive for RSV and SARS-CoV-2; RSV(-)SARS-CoV-2(-), group seronegative for RSV and SARS-CoV-2, BCG, bacillus Calmette–Guérin, SARS-CoV-2, severe acute respiratory syndrome coronavirus 2; RSV, respiratory syncytial virus. The Spearman correlation test was used to assess the correlation of NOD2 mRNA expression and IL-8 in the different study groups. A p-value was considered significant if < 0.05.

**Fig. S8** Correlation between NOD2 receptor expression and IL-8 expression in whole blood cultures stimulated with BCG and SARS-CoV-2 and RSV virus antigens**. Abbreviations**; RSV(+), group seropositive for RSV infection; SARS-CoV-2(+), group seropositive for SARS-CoV-2; RSV(+)SARS-CoV-2(+), group seropositive for RSV and SARS-CoV-2; RSV(-)SARS-CoV-2(-), group seronegative for RSV and SARS-CoV-2, BCG, bacillus Calmette–Guérin, SARS-CoV-2, severe acute respiratory syndrome coronavirus 2; RSV, respiratory syncytial virus. The Spearman correlation test was used to assess the correlation of NOD2 mRNA expression and IL-8 in the different study groups. A p-value was considered significant if < 0.05.

**Fig. S9** Correlation between NOD2 receptor expression and IL-10 expression in whole blood cultures stimulated with BCG and SARS-CoV-2 and RSV virus antigens**. Abbreviations**; RSV(+), group seropositive for RSV infection; SARS-CoV-2(+), group seropositive for SARS-CoV-2; RSV(+)SARS-CoV-2(+), group seropositive for RSV and SARS-CoV-2; RSV(-)SARS-CoV-2(-), group seronegative for RSV and SARS-CoV-2, BCG, bacillus Calmette–Guérin, SARS-CoV-2, severe acute respiratory syndrome coronavirus 2; RSV, respiratory syncytial virus. The Spearman correlation test was used to assess the correlation of NOD2 mRNA expression and IL-8 in the different study groups. A p-value was considered significant if < 0.05.

**Fig. S10** Correlation between NOD2 receptor expression and TNF expression in whole blood cultures stimulated with BCG and SARS-CoV-2 and RSV virus antigens**. Abbreviations**; RSV(+), group seropositive for RSV infection; SARS-CoV-2(+), group seropositive for SARS-CoV-2; RSV(+)SARS-CoV-2(+), group seropositive for RSV and SARS-CoV-2; RSV(-)SARS-CoV-2(-), group seronegative for RSV and SARS-CoV-2, BCG, bacillus Calmette–Guérin, SARS-CoV-2, severe acute respiratory syndrome coronavirus 2; RSV, respiratory syncytial virus. The Spearman correlation test was used to assess the correlation of NOD2 mRNA expression and IL-8 in the different study groups. A p-value was considered significant if < 0.05.
